# Evaluating Post-surgical Stability and Relapse in Orthognathic Surgery: A Comprehensive Review

**DOI:** 10.7759/cureus.72163

**Published:** 2024-10-22

**Authors:** Hanan A Alrashidi, Mohammed H Almutairi, Sarah M Almohaimeed, Lara A Homdi, Aljawhara F Alharbi, Ghadah S Alazmi, Rehab O Mesmeli, Abdullah M Alanazi, Samiyah A Muaini, Kholoud A Alraddadi, Hesham Alowaimer

**Affiliations:** 1 General Dentistry, Ministry of Health, Riyadh, SAU; 2 General Dentistry, Ministry of National Guard - Health Affairs, Riyadh, SAU; 3 Dentistry, Armed Forces Hospital, Riyadh, SAU; 4 General Dentistry, Jazan University, Alkhober, SAU; 5 General Dentistry, Princess Nora Bint Abdulrahman University, Riyadh, SAU; 6 General Dentistry, Hail University, Hail, SAU; 7 General Dentistry, Jazan University, Jeddah, SAU; 8 Dentistry, University of Jordan, Amman, JOR; 9 General Dentistry, Wasfat Dawaa Medical Complex, Jazan, SAU; 10 General Dentistry, Eastern Health Cluster, Al-Nairyah, SAU; 11 Maxillofacial Surgery, Ministry of Health, Dhahran, SAU

**Keywords:** mandibular advancement, maxillary expansion, orthognathic surgery, post-surgical stability, relapse rates

## Abstract

Orthognathic surgery is a procedure that allows oral and maxillofacial surgeons to resolve jaw asymmetry issues and restore function, esthetics, and balance. Orthodontics plays a major part in the pre-surgical and post-surgical phases, thus necessitating a multidisciplinary approach. Certain skeletal discrepancies may remain despite correction with routine growth modification and camouflage treatment, or they may not qualify for these treatments. These skeletal discrepancies are addressed through orthognathic surgeries such as the Le Fort I osteotomy for the maxilla and the bilateral split sagittal osteotomy (BSSO) for the mandible. This narrative review aimed to investigate the factors leading to the instability and relapse of the different surgical procedures by comparing the related literature for all three planes: sagittal, vertical, and transverse. Additionally, it highlights the new trends and modern technology in orthognathic surgery. These findings are targeted at elucidating better surgical approaches, understanding what practices ensure long-term stability, and improving outcomes with greater practitioner and patient satisfaction.

## Introduction and background

Orthognathic surgery and orthodontic treatment are combined procedures indicated for patients with severe dental and skeletal deformities. This combined approach is essential for correcting skeletal misalignments and deranged occlusion [1,2]. A multidisciplinary approach, primarily involving close collaboration between an orthodontist and an oral and maxillofacial surgeon, is required when planning orthognathic surgery. The most commonly performed surgical procedures are the maxillary Le Fort I osteotomy and the mandibular bilateral sagittal split osteotomy (BSSO) for various indications [1]. Surgery becomes an option when growth has ceased, and the patient is no longer a candidate for growth modification or camouflage (including extraction and non-extraction treatments) to correct misaligned dentition and improve overall facial aesthetics [3].

Like any other surgical procedure, there is a risk of relapse with orthognathic surgery, which can impact post-surgical stability. A relapse is any post-treatment change that reverses the outcomes, while post-surgical stability refers to how long the surgical objectives are maintained. To track post-surgical changes, procedures can be categorized as highly stable, with a less than 10% chance of noticeable relapse; fairly or moderately stable, with a less than 20% chance of noticeable relapse and no major post-treatment changes, typically modified with rigid internal fixation, mini-plates, or screws; and least stable, involving significant post-treatment changes [4]. The etiology and causative factors of relapse can vary. Darshan et al. [3] summarized the factors associated with relapse during mandibular advancement, such as the condylar position in the glenoid fossa during internal fixation, a lack of proximal segment control at the time of surgery, para-mandibular connective tissue tension, and advancements greater than 7 mm. However, in the long term, these changes were found to be stable [3].

This review aims to examine in depth the post-surgical stability and relapse following orthognathic surgery, with a focus on different surgical procedures used to address various craniomaxillofacial problems. It seeks to establish a comparison of the factors impacting post-surgical stability, reliability, and predictability of relapse for each procedure. Additionally, new trends and prospects, such as virtual surgical planning and advanced imaging techniques, are discussed in detail to identify new approaches that could improve surgical outcomes, alleviate procedural complications, and enhance the overall patient experience.

## Review

Methodology

This review provides an overview of research findings, spanning from 1996 to 2024, on the factors affecting post-surgical stability and relapse rates across different surgical procedures in all planes. A manual bibliographic search was conducted using PubMed, Google Scholar, Scopus, and ScienceDirect databases with the specified keywords “Post-surgical stability,” “Relapse,” “Orthognathic surgery,” and “Orthodontics.” The extensive literature collected on various surgical procedures, including retrospective studies, systematic reviews, comparative studies, and questionnaire-based studies, was evaluated and analyzed. Only articles published in English, those within the scope of this review, and studies related to different surgical procedures across all three planes were included. Studies published in languages other than English, those addressing non-surgical methods of treatment, and those containing irrelevant information were excluded from the review.

Indications of orthognathic surgery

Among the available treatment options, orthognathic surgery is often considered the last resort. It is typically reserved for cases where the skeletal discrepancy is too severe to be corrected by orthodontics alone or when the patient is no longer growing and cannot undergo growth modification. Many complex cases, such as skeletal class III malocclusion, open bite, and jaw asymmetry, are commonly managed using orthognathic surgery. Andrup et al. [5] conducted a questionnaire survey in Sweden to gather data on the indications and frequency of orthognathic surgeries. The questionnaire was sent to 50 Oral and Maxillofacial Surgery (OMFS) clinics, and 47 clinics responded. The survey revealed that 891 patients across 23 clinics were treated with orthognathic surgery, with functional abnormalities identified as the most important reason for seeking this treatment modality [5].

If facial aesthetics is a significant concern, particularly in cases of severe jaw asymmetry, a surgical approach is often needed. For instance, Peacock et al. [6] conducted a retrospective cohort study of patients who underwent orthognathic surgery. They concluded that for individuals over 40 years of age, the primary motivation for seeking surgical treatment was functional rather than aesthetic. This finding suggested that the motivation for surgery is influenced by age and gender, with more men than women likely to pursue surgery for functional reasons [6]. Some common indications for orthognathic surgery are outlined below, highlighting the necessity of this surgical approach in certain cases.

Aesthetics

Aesthetics is one of the most important indications for orthognathic surgery [7,8]. As the psychosocial well-being of a patient is closely linked to facial harmony and symmetry, careful pre-surgical planning is essential, particularly in the anterior and sagittal directions. This planning should be done through a multidisciplinary approach involving both an orthodontist and a surgeon. In some cases, patients who are satisfied with functional stability after surgery and do not prioritize aesthetics may not require further surgical treatment. In such instances, orthodontic camouflage treatment should be considered as an alternative [5].

Malocclusion

Dental malocclusion can lead to various oral-related diseases and abnormal masticatory function. While dental malocclusion can be treated more accurately through other methods, orthognathic surgery may not offer significant benefits for this particular indication. There is often no substantial improvement, and masticatory function may decrease after surgery as the muscle fibers stretch and the muscles adapt to their new position [9].

Temporomandibular disorders (TMDs)

TMDs encompass a wide range of issues affecting the temporomandibular joint (TMJ), masticatory muscles, head muscles, and neck muscles [10]. There are various widely available options to treat these conditions, with surgical therapy generally considered a last resort after less invasive treatments have been prioritized [11]. Research indicates that between 14% and 97% of patients undergoing orthognathic surgery have TMDs [12,13], but there is limited evidence to suggest that this surgery cures or prevents TMDs [14]. Systematic reviews have shown that symptoms of TMD and joint clicking tend to decrease following orthognathic surgery, while no change is observed in crepitus [12,15]. However, the existing literature does not provide a clear consensus on whether surgery can completely eradicate TMD problems or its overall effectiveness for this purpose, highlighting the need for future studies.

Pre-surgical planning

Patients with skeletal discrepancies often develop natural compensatory adjustments in their teeth to camouflage the discrepancy and maintain functional occlusion [16,17]. This compensation, however, can lead to a non-synchronized relationship between the inclination and position of the teeth, resulting in dentofacial deformity [16]. In conventional orthognathic surgery pre-planning, and pre-surgical orthodontics are done to correct the skeletal discrepancy by decompensating the teeth back to their original positions. This decompensation process allows for better visualization and alignment of the final occlusion with the skeletal correction. The new occlusion then distributes balanced forces across all teeth, achieving occlusal harmony [18]. During the decompensation phase, patients may experience unaesthetic changes in facial appearance and functional instability, but these issues are temporary and part of the preparatory process. Following this phase, surgery is performed, and a brief period of post-surgical orthodontics is often required. As a result, the total treatment duration can extend up to 36 months, and it is important to inform patients of this timeframe beforehand [17, 19].

In contrast, newer techniques, including virtual planning and 3D imaging, allow clinicians to visualize post-surgical changes and predict the procedure's reliability, potentially eliminating the need for pre-surgical orthodontics [2,19,20]. These techniques are discussed in greater detail later in this review.

Post-surgical stability and relapse 

Post-surgical stability is a key measure of a surgical procedure’s effectiveness, as it directly reflects the ability to maintain the desired outcomes over time, whether in the short-term or long-term following surgery [21]. Relapse, defined as the loss of skeletal or dental corrections that were aimed for and achieved during the surgical procedure, is a significant concern and one of the most challenging post-surgical complications to manage [22,23]. The causes of relapse are multifaceted, encompassing both short-term and long-term factors. Short-term relapse may result from condylar dysmorphology, muscular tension due to significant surgical movements, and improper positioning of the condyles in the glenoid fossa. Since these issues can arise almost immediately after surgery, the initial post-operative phase is critical for monitoring and intervention. Long-term relapse, on the other hand, is often associated with more gradual changes, such as chronic progressive alterations in skeletal structure, ongoing skeletal growth, and condylar resorption [24]. For instance, in the study done by Joss et al. [25], the outcomes of mandibular setback (class III) procedures were generally stable in both the short and long term. However, even in these stable cases, certain factors like the extent of the surgical movement, accurate condyle placement, and the patient’s remaining growth potential were crucial in determining the likelihood of relapse [25]. This highlights the importance of meticulous surgical planning and the need for continuous post-operative monitoring to detect and address any signs of relapse early. A dental relapse of ≥2 mm after a follow-up visit following orthognathic surgery is typically considered significant, underscoring the need for precise measurement and ongoing evaluation of the surgical outcomes [26]. 

The factors impacting post-surgical stability and relapse can be broadly classified into pre-surgical, surgical, post-surgical, and patient-related factors.

Pre-surgical Factors

One of the most significant pre-surgical factors affecting long-term stability in orthognathic surgery is the use of pre-surgical orthodontics, which has traditionally been an integral part of the treatment process. To better understand the correlation between pre-surgical orthodontics and post-surgical stability, Kim et al. [27] conducted a study comparing outcomes in patients undergoing mandibular setback for skeletal Class III malocclusion, with and without pre-surgical orthodontics. The study concluded that BSSO without pre-surgical orthodontics (i.e., the surgery-first approach) was less stable than the traditional orthognathic surgery approach, which includes a phase of pre-surgical orthodontics [27]. This finding is crucial because it suggests that while the surgery-first approach may offer benefits in terms of reducing overall treatment time and enhancing patient experience, it may also compromise the stability of the surgical outcome, particularly in patients with complex skeletal discrepancies.

In another evaluation of the surgery-first approach, Peiró-Guijarro et al. [28] expressed concerns about its reliability, particularly regarding long-term stability. They emphasized the need for larger, more comprehensive studies to draw definitive conclusions about the effectiveness and safety of the surgery-first approach [28]. This caution is warranted, as the surgery-first approach eliminates the phase of orthodontic decompensation that traditionally aids in aligning the teeth and jaws in preparation for surgery, thereby potentially increasing the risk of relapse.

Comparative studies have explored the differences in stability between the surgery-first and orthodontics-first approaches to offer insights into the benefits and drawbacks of each method. For example, Akamatsu et al. [29] conducted a retrospective study involving 38 patients with skeletal Class III malocclusion who underwent a sagittal split ramus osteotomy. They found no significant difference in horizontal relapse between the two groups, indicating that both approaches may be similarly effective in maintaining horizontal stability. However, the study found a significant difference in vertical relapse: the surgery-first group experienced a mean vertical relapse of 1.59 mm at the pogonion in the downward direction, compared to 0.14 mm in the upward direction in the orthodontics-first group [29]. This difference suggests that while the surgery-first approach may offer comparable horizontal stability, it may be less effective in controlling vertical stability, particularly in cases where precise control of vertical jaw movements is required. A retrospective study by Valls-Ontañó et al. [30] compared the surgery-first and surgery-late approaches (the latter following pre-surgical orthodontics) in a cohort of 56 patients. The study discovered that while the SNA angle relapsed in both groups, the relapse was more severe in the surgery-late group, particularly among Class III patients [30]. This finding is intriguing; it suggests that even with the additional time and effort invested in pre-surgical orthodontics, the surgery-late approach may not necessarily offer superior long-term stability compared to the surgery-first approach. However, the study also noted that the transverse maxillary dimension remained stable in both groups, highlighting the complexity of factors that influence surgical outcomes and the need for individualized treatment planning.

Surgical Factors (Magnitude of Jaw Movement, Type of Procedure Performed, and Method of Fixation)

Critical determinants of post-surgical stability include the type of surgical technique performed, the direction and magnitude of the surgical movement, the type and material of fixation, condylar repositioning in the glenoid fossa, and the surgeon's experience [31]. These factors do not act in isolation but rather in conjunction with each other to either reinforce or undermine the stability of the surgical outcome. For instance, the choice of fixation material - whether rigid internal fixation (RIF) or other methods - has a significant impact on how well the skeletal structures maintain their new positions post-surgery. The surgeon's expertise in accurately repositioning the condyles within the glenoid fossa is crucial, as any misalignment can lead to condylar resorption or dysfunction, which is a major contributor to long-term instability. Moreover, the magnitude and direction of surgical movements must be carefully planned, as larger movements, particularly in complex cases, inherently carry a higher risk of relapse due to the increased stress on surrounding tissues. In their study on bimaxillary osteotomies, Liebregts et al. [32] highlighted that the occurrence of relapse in double jaw surgeries is a critical concern. They reported a wide range of relapse incidences, from 2.0% to 50.3%, which underscores the variability and complexity of outcomes in such procedures. This relapse can cause the changes achieved through orthognathic surgery to be reversed, thereby exacerbating facial disharmony and dental issues instead of correcting them [32]. The study confirmed that both the amount of surgical jaw movement and the specific surgical techniques employed are significant factors influencing the degree of relapse and post-surgical stability. Larger surgical movements, particularly those involving extensive repositioning of the jaws, impose greater biomechanical stress on the skeletal and soft tissues, which in turn increases the risk of relapse. Additionally, the surgical technique must be meticulously chosen and executed, as even minor deviations can compromise stability, particularly in complex cases involving multiple planes of movement.

A long-term retrospective study by Miao et al. [33] further elucidated the challenges associated with relapse, particularly in patients who had undergone Le Fort I and BSSO with rigid internal fixation for the treatment of skeletal class II malocclusion. Although the maxilla generally remained stable post-surgery, the mandible exhibited a backward movement in the sagittal direction with a relapse of −0.81 ± 1.52 mm at point B. This backward movement, or relapse, led to an increase in the anterior and superior joint space in the TMJ, as detected by MRI, along with a noticeable change in the condylar position [33]. These findings highlight the complex interplay between skeletal movement and joint function, suggesting that even when skeletal alignment is maintained, changes in condylar positioning can introduce instability. This relationship underscores the importance of considering both skeletal and joint dynamics when planning and executing orthognathic surgeries.

The magnitude of hard tissue advancement has been repeatedly identified as a key factor leading to relapse, especially in patients with significant skeletal movements. For instance, Marion et al. [34] conducted a study on 54 patients who underwent a Le Fort I osteotomy (maxillary advancement) for unilateral cleft lip and palate. The study recorded the relapse percentage one year post-surgery and found that the magnitude of maxillary advancement significantly influenced the likelihood of relapse. The rate of horizontal relapse averaged 20.1%, while vertical relapse varied more significantly, with a downward movement relapse rate of 28.4% in some patients and a stability loss of 7% in others who experienced upward movement [34].

The variability in these relapse rates highlights the sensitivity of the maxilla to surgical movements, particularly in patients with pre-existing conditions such as unilateral cleft lip and palate. The study also emphasized a threshold value of 7 mm for maxillary advancement, beyond which the risk of relapse increases substantially [34]. This finding is crucial for surgical planning, as it suggests that while larger advancements may be necessary for functional and aesthetic reasons, they also pose a higher risk of relapse if not carefully managed. The interplay between the magnitude of surgical movement and the stability of the outcome necessitates a cautious approach, particularly in complex cases where the risk of relapse is inherently higher due to the extent of the surgical intervention.

The choice of procedure to address a specific problem is crucial in predicting the post-surgical outcomes of orthognathic surgery performed for a particular skeletal deformity. Ito et al. [35] studied 18 cases of skeletal open bite malocclusion and theorized that if the condition is only treated at the mandibular level with upward repositioning in an anti-clockwise direction, without sufficient mandibular backward repositioning, it could cause the corrected open bite to relapse [35].

Double jaw surgeries are as common as isolated jaw surgeries. When Proffit et al. [31] compared stability in Class III patients undergoing combined mandibular setback and maxillary advancement surgeries with isolated procedures; they found no significant difference in stability between the two approaches, with equivalent results for each jaw [31]. Similarly, a study by Kim et al. [36] confirmed the stability and predictability after two-jaw surgeries. Their study examined records of 12 male and 14 female patients through CBCT before surgery, after surgery, and at follow-up. Although changes such as inward rotation of the coronal condyle axis and a shift in the anteroposterior position of the condyle toward a concentric position were observed, indicating a relapse of position, these changes did not adversely affect stability after double jaw surgery [36].

Arpornmaeklong et al. [37] investigated the stability of bi-maxillary surgical procedures with rigid internal fixation for the treatment of skeletal Class II malocclusion. They evaluated the combined Le Fort I maxillary impaction and mandibular advancement performed in 29 patients, with a mean age of 22.6 years, using serial lateral cephalograms taken during a one-year follow-up period. The maxillary impaction proved to be stable, with no significant changes observed at follow-up. However, a notable mandibular relapse was recorded in five female patients who were high-angle cases and underwent mandibular advancement of greater than 10 mm. They concluded that while the majority of patients remained stable, a small number experienced a skeletal relapse in the mandible, primarily due to condylar resorption and remodeling [37].

Mishra et al. [38] also evaluated the success of bi-jaw orthognathic surgery, focusing on the anterior segments operated on for dentoalveolar protrusion. The results were obtained by assessing changes in hard and soft tissues in the vertical and horizontal dimensions before and after surgery. They found that out of 20 cases analyzed through manual tracing of lateral cephalograms, there was insignificant relapse and minimal post-operative complications, making it a simple procedure with high levels of patient satisfaction [38]. Liebregts et al. [32] explored a new concept regarding the stability of orthognathic surgery, specifically whether performing maxilla-first or mandible-first surgery would yield long-term stable outcomes. This one-year follow-up study, involving 106 patients divided equally into two groups, used the OrthoGnathic Analyzer to assess virtually pre-planned translations and rotations. The results indicated that the average anteroposterior, transverse, and vertical relapse was less than 1.8 mm, with no significant difference between maxilla-first and mandible-first surgeries. Although further research is needed, this study successfully demonstrated the excellent role of virtual 3D planning in ensuring the long-term stability and predictability of outcomes [32].

The type of fixation used also impacts procedural stability, as highlighted by Masson et al. [39] in their study on rigid osteosynthesis with titanium plates and monocortical screws for genioplasty advancement. They found that rigid osteosynthesis provided long-term stability, with minimal relapse and bone remodeling induced by surgery. Relapse was recorded as 0.65 mm in the sagittal plane and 0.34 mm in the vertical plane. Additionally, they noted that relapse was not influenced by gender, age, or degree of bone advancement [39]. Lai et al. [40] studied mandibular surgery involving two fixation methods (unilateral plates or bilateral plates) and analyzed the post-surgical relapse rates. They found no significant difference in the outcome, and long-term stability results were positive for both fixation methods. However, they identified pre-operative condylar bony changes as a factor that might increase the risk of relapse in those patients [40]. Buch et al. [41] investigated single-jaw (maxillary) surgery and bi-jaw surgery using three bi-cortically fixated screws to ensure long-term stability. They inferred that there was a greater need for stability, as the already existing auxiliaries were insufficient for anterocranial stability [41].

Post-surgical Factors

One major post-surgical factor is post-surgical orthodontics, which typically involves a brief period to finalize the occlusion and thereby enhance long-term stability. However, Ito et al. [35] studied the treatment of skeletal anterior open bite malocclusion by repositioning the mandible upward in a counter-clockwise rotation and found that it was crucial to avoid stimulating the extrusion of molars. This approach helped preserve the stability of the open bite correction, thus negating the need for post-surgical orthodontics [35]. Therefore, the timing and sequence of orthodontic treatment phases must be strategically planned to balance immediate outcomes with long-term stability.

Patient-Related Factors

Navarro-Fernández et al. [42] investigated the patient-related factors that influenced post-surgical outcomes in orthognathic procedures. They found that patients' psychological states, particularly their pre-surgical expectations and anxiety, can significantly impact their recovery and overall satisfaction with the results. The study found that high levels of anxiety and specific fears related to the procedure, such as kinesiophobia (fear of movement), could lead to poorer post-surgical outcomes. Pre-surgical kinesiophobia, in particular, showed a correlation with post-surgical kinesiophobia, suggesting that patients who are anxious about moving post-surgery may experience prolonged recovery times or complications due to reduced mobility and muscle stiffness [42]. This finding underscores the importance of addressing psychological factors during the pre-surgical phase, as they can have a profound impact on patient adherence to post-operative care protocols and, ultimately, the success of the surgery. It also highlights the need for a multidisciplinary approach that includes psychological support alongside medical and surgical care, ensuring that patients are mentally and emotionally prepared for the procedure.

There is a wide range of biological and behavioral aspects that can significantly influence the outcome of surgery. Factors such as masticatory function, interdigitation, neuromuscular adaptation, patient age, skeletal pattern type, response to global growth, abnormal behavior of the orofacial musculature, and pressure from adaptive posturing of the tongue play crucial roles in determining stability. For example, younger patients with ongoing skeletal growth may experience continued changes that challenge the stability of surgical outcomes. Additionally, the neuromuscular adaptation post-surgery is critical - if the muscles fail to adapt properly to the new jaw position, they can exert forces that gradually lead to a relapse. The behavior of orofacial muscles, such as tongue thrusting or atypical swallowing, can also place continuous pressure on the surgical site and further contribute to instability. Understanding these patient-specific factors is essential for tailoring both the surgical and post-surgical treatment plans to enhance stability. Table 1 summarizes the factors.

**Table 1:** Factors influencing post-surgical stability and relapse in orthognathic surgery

Factor category	Specific factors
Pre-surgical factors	1-Pre-surgical orthodontics improves long-term stability [27].
	2-Surgery-first approach may reduce treatment time but potentially increases the risk of relapse in complex cases [27-30].
Surgical factors	1-Type and magnitude of jaw movement (greater than 7 mm increases relapse risk) [34].
	2-Condyle positioning in the glenoid fossa [31].
	3-Method of fixation, such as rigid internal fixation (RIF), crucial for maintaining skeletal positions [31, 32].
	4-Surgeon’s expertise is key for accuracy and long-term stability [31].
Post-surgical factors	1-Post-surgical orthodontics to finalize occlusion and reduce the likelihood of relapse [35].
	2-Initial post-operative phase is critical for detecting short-term relapse [24].
Patient-related factors	1-Psychological state (anxiety, expectations) affecting recovery and adherence to post-op care [42].
	2-Neuromuscular adaptation, particularly muscle adaptation to new jaw positioning [42].
	3-Orofacial muscle behavior (e.g., tongue thrusting, atypical swallowing) may apply continuous pressure on the site [42].

What to expect after surgery? Short-term stability versus long-term stability

The primary goal of orthognathic surgical procedures combined with orthodontic treatment is to achieve both dental and skeletal stability. This dual focus on stability is essential for maintaining both the functional and aesthetic outcomes of the surgery over time. Post-surgical stability is generally categorized into two distinct phases: short-term and long-term stability. According to Proffit et al. [26], in their comprehensive study on the hierarchy of stability, short-term stability refers to the functional adaptation of hard tissues observed during the initial healing phase following surgery and orthodontic treatment. In contrast, long-term stability encompasses changes monitored over an extended period, influenced by a complex interplay of factors such as surgical technique, orthodontic interventions, and patient-specific variables [26]. The distinction between short-term and long-term stability is crucial because each phase is governed by different biological processes and clinical challenges. Short-term stability is primarily concerned with the immediate post-operative period, where the surgical outcomes are solidified, and the tissues begin to adapt to their new positions. This phase is critical for setting the foundation for long-term success, as any early shifts or instability could compromise the overall treatment objectives.

The existing literature is particularly rich in studies that examine short-term (0.5 to two years) post-surgery dental stability. These investigations provide valuable insights into the initial success of orthognathic procedures and identify potential early complications or relapses. However, achieving long-term stability has always been the ultimate goal following orthognathic surgery and orthodontic treatment, as it reflects the enduring success of the intervention [1]. Long-term stability is influenced by a broader range of factors, including ongoing skeletal growth, neuromuscular adaptation, and the potential for late-onset changes in dental occlusion or jaw positioning.

For instance, Hoffman et al. [43] conducted a study on the stability of mandibular advancement in patients treated with BSSO and subsequent orthodontic treatment. They analyzed both short-term (six weeks post-surgery) and long-term (12 months post-surgery) horizontal stability in 15 patients. The study reported a mean advancement of 6.1 mm, with minimal continued movement (0.16 mm) observed after six weeks and a long-term relapse of 0.46 mm. These findings suggest that while some degree of minor adjustment occurs shortly after surgery, the long-term stability is largely maintained, particularly when bicortical screw fixation is used in BSSO [43]. This conclusion is significant because it highlights the importance of the fixation method in ensuring stable outcomes, reinforcing the pivotal role of surgical technique in both short-term and long-term stability.

**Figure 1:**
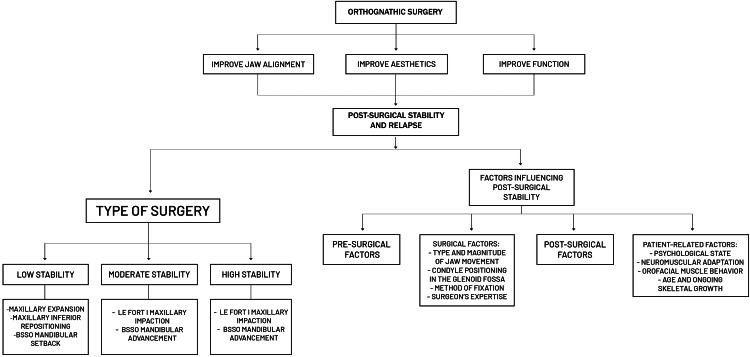
Orthognathic surgery: stability, relapse, and influencing factors BSSO: Bilateral Sagittal Split Osteotomy

Moreover, Mulier et al. [1] specifically focused on the long-term stability of dental and maxillofacial changes over a five-year post-operative follow-up period. Their study revealed significant variability in the stability of skeletal Class II and Class III malocclusions, underscoring the challenges in predicting long-term outcomes [1]. This variability emphasizes the importance of individualized treatment planning and the need for continuous monitoring using advanced imaging techniques, such as computed tomography (CT) or cone-beam CT (CBCT). These imaging modalities allow for precise assessment of bone and soft tissue changes, reducing the risk of bias in retrospective studies and providing more definitive insights into the factors that influence long-term stability. The findings from Mulier et al. [1] also suggest that relying solely on short-term data may not provide a complete picture of the treatment's success. Long-term studies are essential to capture the full spectrum of changes that can occur years after the initial surgery, including the effects of aging, ongoing skeletal development, and patient compliance with post-surgical orthodontic care. By integrating long-term follow-up into the standard of care, clinicians can better anticipate potential issues and implement strategies to mitigate them, ultimately leading to more predictable and stable outcomes for patients. Figure 1 shows stability factors and hierarchy.

Comparative analysis of stability and relapse in different surgical procedures

Surgical corrections in orthognathic surgery are performed across all three planes of orientation: sagittal (anteroposterior), vertical, and transverse. Below is a detailed discussion on the correction of the most common problems encountered in orthodontics and OMFS, along with their post-surgical stability.

Anteroposterior corrections 

Le Fort I: Maxillary Advancement

Saltaji et al. [44] investigated the outcomes of the Le Fort I osteotomy in patients with cleft lip and palate, specifically targeting the correction of maxillary hypoplasia. This procedure is critical in addressing the underdevelopment of the midface, which can severely affect both function and aesthetics. To assess the success and predict the stability of the procedure, the systematic review compiled data from various studies and evaluated relapse rates observed over a follow-up period of one year or more. The findings indicated that horizontal relapse at point A ranged from 20% to 30% in four studies, while three studies reported a higher horizontal relapse rate of 30% to 40%. Vertical relapse, however, was even more pronounced, with more than 50% reported in four studies [44].

These results suggested that while a Le Fort I osteotomy can effectively address maxillary hypoplasia, the procedure is associated with a moderate rate of horizontal relapse and a considerably higher rate of vertical relapse [44]. The significant vertical relapse highlights the challenge of maintaining vertical stability in cleft lip and palate patients, possibly due to the complex interplay of forces in the vertical dimension, such as muscle tension, occlusal forces, and growth factors. This underscores the need for detailed surgical planning and postoperative management, especially in controlling vertical dimensions to ensure long-term stability.

In contrast, Wangsrimongkol et al. [45] conducted a similar retrospective study focusing on the Le Fort I advancement procedure in cleft lip and palate patients but obtained different results. The study categorized patients into three groups (mild, moderate, and severe) based on the severity of maxillary hypoplasia according to Wits analysis. Lateral cephalograms were used to measure changes at three key points-before surgery (T1), immediately after surgery (T2), and at the one-year follow-up (T3). The study found that the mean relapse ranged between 1 and 1.5 mm across all groups, with no significant difference observed at the one-year mark, regardless of the severity of the initial hypoplasia [45].

These findings suggest that the Le Fort I osteotomy can be a stable procedure for cleft lip and palate patients, particularly when overcorrection is employed to compensate for anticipated relapse. The use of overcorrection in surgical planning is a strategic approach, especially in cases where minor post-surgical adjustments are expected [45]. The study’s conclusion that moving the maxilla forward is relatively stable highlights the effectiveness of this technique in addressing maxillary hypoplasia, though it also points to the importance of individualized treatment plans and the potential benefits of long-term follow-up to monitor and ensure the reliability of outcomes.

Le Fort I: Maxillary Setback

A maxillary protrusion, often seen in skeletal Class II malocclusion, can be effectively corrected through maxillary setback procedures. Xiang et al. [46] explored various methods for achieving this correction and compared the outcomes of a Le Fort I osteotomy performed with either maxillary tuberosity removal or intentional pterygoid plate fracture (IPPF). This study included 80 patients who underwent double jaw surgery (Le Fort I osteotomy and BSSO) and divided the patients into two groups: Group I patients were treated with maxillary tuberosity removal and Group II patients were treated with IPPF. After a one-year follow-up, both groups demonstrated stable results; however, the IPPF group had several advantages, including less blood loss and a shorter operative time [46].

The comparison between these two techniques provides valuable insights into the optimization of surgical approaches for maxillary setbacks. The advantages observed with IPPF, such as reduced blood loss and operative time, suggest that it may be preferable in certain clinical scenarios. The stability achieved in both groups reinforces the idea that carefully executed maxillary setbacks can lead to reliable outcomes. However, when choosing between techniques, the clinician should consider not only the immediate surgical benefits but also long-term stability and the potential for relapse [46]. Rotational maxillary setback has also been studied as a treatment modality for skeletal Class III malocclusion, particularly in patients with proclined incisors and/or a protrusive maxilla. This approach was found to be stable, especially when evaluating skeletal landmarks such as the posterior nasal spine (PNS), which remained stable both vertically and in the anteroposterior plane. However, the study did note some dental instability, which was associated with a higher risk of relapse and needed subsequent orthodontic correction [47].

The observation of dental instability in rotational maxillary setback highlights a critical aspect of post-surgical care: the need for vigilant monitoring and potential orthodontic intervention to manage any dental shifts that could compromise the overall stability of the procedure. While skeletal landmarks may remain stable, the dental components can be more susceptible to minor movements that could lead to functional and aesthetic concerns if left unaddressed.

Bilateral Split Sagittal Osteotomy (BSSO): Mandibular Advancement

BSSO is a commonly performed procedure to correct mandibular retrognathism in Class II malocclusion, where the mandible is positioned too far back relative to the maxilla. This procedure aims to advance the mandible to improve both function and aesthetics. Paunonen et al. [48] conducted a study to evaluate the relapse rate of BSSO and identify factors that contribute to its success or failure. The study found that the mean mandibular advancement was 5.7 mm, with an average relapse of 0.1 mm based on overjet findings and a post-surgical corrected overbite relapse of 1.1 mm. Interestingly, the study observed that approximately 25% of the advancement relapse occurred at the Co-Gn measurement (condylion to gnathion), a crucial indicator of skeletal stability. Despite this, factors such as the patient's age, gender, and the surgeon's experience did not significantly impact the relapse rate. This finding suggests that BSSO is inherently stable for correcting mandibular retrognathism in Class II malocclusion patients, with the 25% relapse attributed to clinically non-significant dental discrepancies that occur over time [48]. The study’s results are particularly valuable because they underscore the robustness of BSSO as a surgical technique. Even with some degree of relapse, the impact on overall treatment outcomes is minimal, implying that BSSO provides a reliable correction for mandibular retrognathism. This stability is crucial for patient satisfaction and long-term functional outcomes, as it ensures that the improvements gained through surgery are maintained over time.

Mandibular advancement is also indicated in patients with idiopathic condylar resorption (ICR), a condition characterized by progressive deterioration of the mandibular condyle and associated bone loss without a known etiology. ICR often leads to bilateral skeletal Class II malocclusion, which is characterized by a retruded mandible, high mandibular plane angle, and anterior open bite. Patients with ICR may attempt to compensate for the deformity by posturing the mandible forward, resulting in muscular dysfunction and further complicating treatment. These patients typically undergo a combination of functional splint therapy, orthognathic surgery, and orthodontic treatment. In a study focusing on patients with this condition, 13 out of 16 experienced minimal relapse (less than 2 mm), and only two showed more than 2 mm of mandibular setback. The study concluded that functional splint therapy increases the reliability and stability of the surgical procedure by promoting neuromuscular adaptation and supporting the newly positioned mandible [49]. This finding is significant as it highlights the importance of a multidisciplinary approach in managing complex cases like ICR. The combination of functional therapy and surgical intervention appears to enhance the stability of mandibular advancement, suggesting that addressing the underlying functional issues is as important as the surgical correction itself. This integrated approach not only increases post-surgical stability but also enhances patient outcomes by reducing the risk of relapse.

Telha et al. [50] further investigated the stability of BSSO performed for mandibular advancement by comparing hybrid and non-hybrid rigid internal fixation techniques. The study divided seventy adult patients with skeletal Class II deformity into four groups and analyzed the impact of different fixation methods on relapse rates. While the study primarily focused on the fixation technique, it found that all four groups remained fairly stable, with an irrelevant relapse of less than 2 mm. However, the study also highlighted that the magnitude of mandibular advancement plays a significant role in the long-term stability of the procedure [50]. This observation reinforces the idea that while fixation methods are important, the extent of surgical advancement itself is a critical factor that determines long-term stability. Larger advancements inherently impose higher risks of relapse due to the increased biomechanical forces acting on the repositioned mandible. Therefore, careful planning and execution are essential, particularly in patients requiring significant mandibular advancement.

Overall, it has been consistently demonstrated that mandibular advancement via BSSO is a highly stable procedure, with the degree of advancement being a key factor influencing the relapse rate. This stability is essential for ensuring long-term success and patient satisfaction, as it minimizes the need for additional corrective procedures or extensive post-surgical orthodontics.

BSSO: Mandibular Setback

Mandibular setback surgery is performed to correct mandibular prognathism, a condition where the mandible is positioned too far forward relative to the maxilla, resulting in facial disharmony and functional issues. While this procedure is generally considered stable in the long term, various studies have called its predictability into question. For instance, a comparative study by Darshan et al. [3] investigated the long-term stability of mandibular setback versus mandibular advancement surgeries. The study found that while mandibular advancement had a relapse rate of 7%, mandibular setback exhibited a significantly higher relapse rate of approximately 29%, making it a less stable procedure [3]. The higher relapse rate associated with mandibular setback is concerning, as it suggests that this procedure is inherently more prone to instability. The reasons for this increased instability may include the anatomical and biomechanical challenges of repositioning the mandible backward, which can disrupt the balance of forces within the masticatory system. This disruption may lead to changes in muscle tension and generate occlusal forces that contribute to relapse over time.

Further supporting this notion, Luchi et al. [51] conducted a study using cephalometric landmark comparisons before and after surgery to investigate the relapse rate in mandibular setback procedures. The study involved 20 adult male patients with skeletal Class III malocclusion who underwent pre-operative orthodontic treatment followed by surgery and fixation. The results showed that relapse was more pronounced in the horizontal plane, with Point B exhibiting up to 37.75% relapse and Point Pg showing 45.85% relapse. The study suggested that the improved adaptation of musculature and intercuspation led to an anticlockwise rotation of the mandible, thus contributing to forward relapse [51].

This finding highlights a critical issue with mandibular setback: the potential for significant skeletal relapse, particularly in the horizontal plane. The study’s suggestion that muscle adaptation and occlusal changes can induce a rotational relapse underscores the complexity of achieving stable outcomes in these cases. It also suggests that additional measures, such as muscle retraining or targeted orthodontic interventions, may be necessary to preserve the surgical outcomes and prevent relapse. To control relapse after mandibular setbacks, Kim et al. [52] studied the use of bioabsorbable plates (poly-I/dl-lactide plate system - Biosorb FX (Linvatec Biomaterials Ltd, Tampere, Finland)) in 30 patients who underwent BSSO. The use of bioabsorbable plates improved stability in the horizontal direction, but significant relapse was observed in the vertical direction due to increased vertical dimension [52]. This finding indicates that while bioabsorbable plates may offer benefits in certain dimensions, they do not eliminate the risk of relapse, particularly in the vertical plane. This suggests that fixation techniques and materials need to be further refined to enhance overall stability.

Another study compared bi-cortical (BCO) and mono-cortical osteosynthesis (MCO) in BSSO for the correction of mandibular prognathism. The study divided patients into two groups: 25 received BCO, and 32 received MCO. The results showed that the sagittal relapse rate was 20% in the BCO group and 25% in the MCO group, primarily due to the forward-upward rotation of the mandible. Due to the lack of significant difference between the sagittal and vertical changes in the two groups, the researchers concluded that there was no substantial difference in post-surgical stability between the two fixation methods [53]. This result suggests that the choice between BCO and MCO may not be as critical as once thought, at least in terms of sagittal and vertical stability. However, it also underscores the persistent challenge of controlling rotational movements and preventing relapse, regardless of the fixation method used.

Hågensli et al. [54] conducted a study to assess the long-term stability of BSSO for mandibular setbacks and also to investigate potential nerve injury as a procedural complication. The study found that the procedure generally showed long-term stability, with improvements in chin asymmetry by an average of 56% and skeletal relapse in the sagittal and transverse planes at 10%-15%. However, 58% of patients experienced relapses of about 3 mm at the mention during a three-year follow-up visit. Despite these relapses, patient satisfaction and acceptance of the procedure were high, with dental midline shifts improving in 80% of cases. The study also noted that the risk of sensory impairment was present, particularly in cases involving distal segment rotation during surgery on the deviating side [54]. The findings from Hågensli et al. [54] further emphasize the complexity of mandibular setback procedures. While they can provide significant improvements in aesthetics and function, the risk of relapse and complications such as nerve injury must be carefully considered. The high rate of patient satisfaction despite these issues suggests that the benefits of the procedure often outweigh the drawbacks, but it also highlights the need for thorough patient counseling and individualized treatment planning [54]. To further complicate stability outcomes, condylar sagging - the downward displacement of the mandibular condyle following surgery may also play a role in postoperative relapse. This displacement can alter the occlusal alignment and contribute to TMJ dysfunction, affecting both aesthetics and function [51-53]. Managing condylar positioning during BSSO is crucial to minimize these risks, especially in complex cases involving rotational movements [51-53]. Future research is needed to explore the relationship between condylar sagging and long-term stability, providing insights into strategies for improving surgical outcomes.

The research consensus indicates that mandibular setback is less stable than other orthognathic procedures, and its stability is not significantly improved by different fixation methods. This underscores the inherent challenges of mandibular setback, where the biomechanical forces involved make it difficult to achieve long-term stability. Continued research and innovation in surgical techniques, fixation materials, and post-operative care are essential to improve the predictability and outcomes of mandibular setback surgeries.

Vertical corrections

Maxillary impaction (superior repositioning) and inferior repositioning are surgical procedures used to correct maxillary vertical deficiency or excess. These procedures are commonly employed to treat patients presenting with deep bite or open bite. Numerous studies have examined the long-term stability of these orthognathic procedures.

Maxillary Impaction

Antonarakis et al. [55] studied cases of myotonic dystrophy, a neuromuscular disorder that leads to significant oro-facial muscle weakness and a lowered tongue position. It often results in vertical excess and anterior open bite in affected patients. Managing these cases is particularly challenging due to the combination of weakened masticatory function and the predisposition to vertical skeletal discrepancies. The study used pre-surgical orthodontics followed by carefully planned surgical interventions. However, despite these efforts, the long-term outcomes were not favorable. The instability observed was largely due to the extrusion of posterior teeth, weakened masticatory function, and remodeling and repositioning of the TMJ. These factors contributed to the high rate of relapse, undermining the surgical corrections made. The study concluded that if specific factors, such as TMJ remodeling and the impact of muscular dysfunction, are carefully considered, and treatment is delayed until a later age when growth is more stable, more stable and acceptable outcomes could be achieved [55]. This conclusion is significant because it underscores the importance of individualized treatment planning in patients with complex conditions like myotonic dystrophy. The need for permanent retention and a tailored approach that accounts for the unique challenges posed by the condition highlights a key principle: successful orthognathic surgery is not solely about the surgical technique but also about timing and the management of underlying systemic conditions.

Solano-Hernández et al. [56] also explored cases of maxillary excess and skeletal open bite by comparing different surgical approaches to address vertical excess and evaluating their long-term stability. The study found that after a Le Fort I osteotomy, there was a notable long-term relapse in dental overbite. Furthermore, when the procedure was combined with bi-maxillary surgery, the relapse was even more significant, particularly in the mandibular plane angle and inter-maxillary angles. Additionally, the study observed greater relapse in anterior facial height following bi-maxillary surgery compared to Le Fort I osteotomy alone. These findings led to the conclusion that bi-maxillary surgery results in greater vertical instability, which is a critical consideration when planning for long-term stability in such patients [56]. The greater relapse observed in bi-maxillary surgery suggests that while this approach might be necessary for certain severe deformities, it carries an increased risk of vertical instability. This could be due to the complexity of coordinating movements in both jaws, which may introduce additional vectors of force that contribute to relapse. Therefore, clinicians must weigh the benefits of initial correction through bi-maxillary surgery against the potential for increased instability and plan accordingly to mitigate these risks.

Similarly, Torgersbråten et al. [57] addressed the challenges of managing Class II malocclusion cases with a high vertical angle through double jaw surgery, which included a Le Fort I maxillary osteotomy and mandibular BSSO. These cases are particularly complex due to the increased risk of relapse, which the study identified as a significant concern. The study involved a three-year follow-up of 36 cases, 13 of which included genioplasty. The results indicated that 40% of patients experienced a mandibular relapse of 2 mm or more. The study concluded that advancements greater than 10 mm significantly increased the risk of horizontal relapse. Additionally, half of the patients experienced bite opening, although maxillary movement and overjet remained stable. Interestingly, the inclusion of genioplasty did not significantly impact the relapse outcome, suggesting that other factors, such as the magnitude of advancement and the vertical angle of the occlusal plane, played a more critical role in determining stability [57]. The findings from this study highlight the need for careful surgical planning and consideration of the extent of jaw advancement in patients with high vertical angles. The fact that genioplasty did not significantly affect relapse rates suggests that the primary factors influencing stability in these cases are the magnitude of surgical movement and the patient’s unique anatomical characteristics. This emphasizes the importance of individualized treatment plans and the potential need for conservative advancements to minimize relapse risk.

Teittinen et al. [58] examined the impact of double jaw surgery versus isolated superior repositioning of the maxilla on long-term stability in correcting open bite cases. The study divided 24 patients into two groups: one undergoing maxillary impaction only and the other undergoing maxillary impaction combined with mandibular osteotomy. Lateral cephalograms were taken before surgery, immediately after surgery, and at a three-year follow-up. The follow-up results showed a moderate relapse of the maxilla in the vertical plane, with the mandible showing relapse in both vertical and sagittal planes, particularly after double jaw surgery. The study found that patients with high vertical angles and sagittal discrepancies were more vulnerable to relapse, indicating the need for further prospective studies with larger sample sizes to investigate potential risk factors and ensure long-term stability [58].

This study underscores the challenges in achieving long-term stability in cases involving both vertical and sagittal corrections. The observation that patients with high vertical angles and sagittal discrepancies are more prone to relapse suggests that these factors must be carefully evaluated during treatment planning. Additionally, moderate relapse has been observed even in cases of isolated maxillary impaction, indicating that vertical control remains a significant challenge in orthognathic surgery. This is particularly true in complex cases involving multiple planes of correction [58]. Although Proffit et al. [31] regarded superior repositioning of the maxilla as one of the most stable procedures, subsequent research has uncovered interesting phenomena surrounding this technique. Specifically, the long-term stability of maxillary impaction appears to be at higher risk when performed as part of bi-maxillary surgery. This finding emphasizes the need for long-term follow-up visits to monitor stability and promptly address any emerging issues during the post-operative period [31].

The contrast between the perceived stability of maxillary impaction and the challenges observed in practice highlights the importance of ongoing research and clinical vigilance. While superior repositioning of the maxilla may be stable in isolated cases, the addition of other surgical interventions, such as mandibular osteotomies, can introduce new variables that complicate the long-term stability of the procedure. This reinforces the importance of comprehensive follow-up care and potentially using adjunctive therapies or interventions to maintain surgical outcomes.

Maxillary Inferior Repositioning

While maxillary impaction is commonly employed to treat open bites, maxillary inferior repositioning is a specialized treatment used for deep bite cases, particularly in patients with vertical insufficiency. This procedure involves repositioning the maxilla downward to correct the vertical dimension of the face, thereby improving both facial aesthetics and function. The goal is to create a more balanced facial profile while addressing functional issues related to occlusion and bite.

Santos et al. [59] conducted a study to investigate the long-term stability of maxillary inferior repositioning, focusing on cases where fixation was achieved using four 2.0 mm L-shaped mini plates without the use of bone grafts. The study used lateral cephalograms to assess linear movements at critical anatomical points, including the anterior nasal spine, PNS, point A, the top of the incisor, and the buccal-mesial cusp of the first molar. After a follow-up period of six months, the study discovered that this method of internal rigid fixation without grafts was notably unstable. The results indicated that the maxillary inferior repositioning procedure was not a reliable long-term solution for patients with maxillary insufficiency. It was regarded as the most unstable procedure among those evaluated [60]. The instability observed in this study is significant because it highlights the challenges of managing vertical corrections in deep bite cases. The lack of bone grafting, which might have provided additional structural support, likely contributed to the observed instability. The findings suggest that while maxillary inferior repositioning can initially correct vertical insufficiency, the absence of adequate stabilization techniques, such as grafting, leads to significant relapse and can undermine surgical outcomes. Convens et al. [60] corroborated these findings and identified a consistent pattern of relapse associated with maxillary inferior repositioning and rigid fixation. Their study recorded an absolute relapse of a mean of 1.6 mm in the anterior maxilla and 0.3 mm in the posterior maxilla after six months of treatment. These measurements reflect a considerable degree of post-surgical movement, particularly in the anterior maxilla, which is critical for maintaining the intended facial and occlusal outcomes [60].

The data from these studies [59,60] suggest that maxillary inferior repositioning, particularly without the use of bone grafts or additional stabilization techniques, is prone to significant relapse. This makes it a less reliable option for long-term correction of vertical insufficiency and highlights the inherent challenges of achieving stability in vertical repositioning procedures. The observed differences in relapse between the anterior and posterior maxilla may be attributed to the various biomechanical forces at play in these regions, further complicating the predictability of outcomes.

Due to the instability of the maxillary inferior repositioning discussed above, various alternative fixation methods have been explored to improve surgical outcomes. However, these methods have had limited success, indicating that the problem is not solely related to fixation techniques but is also due to the inherent challenges of the procedure itself. The findings emphasize the need for a deeper understanding of the factors influencing stability in these cases, including patient-specific factors such as bone density, facial morphology, and muscle function, which may all influence the variability of outcomes. Given the challenges and high relapse rates associated with maxillary inferior repositioning, a series of long-term studies are necessary to investigate the nuances of this surgical procedure further.

Transverse corrections

*Maxillary Expansion* A narrow maxillary arch, often seen in cases of cleft lip and palate, indicates a significant discrepancy in the transverse plane of the face. This condition not only affects facial aesthetics but also compromises occlusal function, leading to issues such as anterior open bite and posterior crossbite. Maxillary expansion is one of the most common and well-researched orthognathic procedures used to correct transverse maxillary deficiency. Surgically Assisted Rapid Maxillary Expansion (SARPE) is particularly indicated for adult patients whose palatal sutures have yet to be fused, making traditional orthodontic expansion methods less effective.

Takeuchi et al. [61] conducted a case study on an 18-year, 7-month-old male patient with a narrow maxilla, anterior open bite, and bilateral posterior crossbite caused by the transverse discrepancy. The treatment plan involved the maxillary expansion of greater than 8 mm, using SARPE combined with Le Fort I corticotomy and BSSO for the mandibular setback. The total treatment duration was 24 months. Post-treatment outcomes showed a successful Class I molar relationship, with maxillary expansion of 6.3 mm at the level of the canines and 9.7 mm at the level of the first molars. Remarkably, the relapse rates after two years were minimal, at 0.9 mm and 0.1 mm, respectively. The study concluded that SARPE is an effective and reliable procedure for addressing maxillary transverse deficiencies. However, it also acknowledged the need for further long-term studies to confirm these findings across a broader patient population [61].

This case highlights the potential of SARPE to provide substantial and stable transverse expansion, even in complex cases involving severe discrepancies [61]. However, it is important to acknowledge that case reports alone are not sufficient to draw definitive conclusions on the long-term stability of SARPE outcomes. The minimal relapse observed suggests that SARPE, when combined with appropriate surgical adjuncts such as Le Fort I corticotomy, may offer promising long-lasting results [61]. The study also emphasizes the importance of ongoing monitoring and the potential need for individualized retention strategies to ensure the longevity of the correction [61].

Despite the generally favorable outcomes associated with SARPE, the existing literature underscores the importance of using auxiliary devices and retention strategies to maintain long-term stability. Gonga et al. [62] emphasized the critical role of retention devices and distractors in improving post-surgical stability. Their research indicates that the careful selection and application of these devices can significantly reduce the risk of relapse, thereby ensuring that surgical corrections are maintained over time [62]. This finding is particularly important given the dynamic forces acting on the expanded maxilla, which, without adequate support, could lead to a gradual return to the pre-surgical state.

Furthermore, Galli et al. [63] investigated the use of Patient-Specific Fixation Implants (PSFI) in combination with SARPE, but without the use of traditional intraoral retention. They found that this approach not only enhanced surgical accuracy but also improved post-treatment stability. The custom-designed implants provided precise and stable fixation, reducing the reliance on conventional retention methods and demonstrating the procedure's overall reliability [63]. This innovation in fixation technology represents a significant advancement in orthognathic surgery, particularly for patients requiring substantial transverse expansion.

The combined insights from these studies suggest that while maxillary expansion is not inherently stable, its predictability and long-term success can be significantly enhanced using various fixation devices and advanced surgical techniques. Compared to more invasive procedures like the Le Fort I osteotomy, SARPE specifically appears to offer a more controlled and stable outcome, particularly when augmented with modern fixation methods and tailored retention strategies [31]. These findings reinforce the importance of a tailored approach for each patient, utilizing the latest advancements in surgical technology and post-operative care to maximize the stability and effectiveness of maxillary expansion.

Mandibular Expansion

Mandibular transverse deficiency, while common, has not been as extensively studied as maxillary deficiencies, particularly in the context of orthognathic surgery. This may be due to the greater prevalence of maxillary issues or the complexities associated with mandibular surgical procedures. Nonetheless, addressing mandibular transverse deficiencies is crucial for achieving balanced occlusion and overall facial harmony. The two primary surgical methods employed for mandibular expansion are corticotomy and mandibular symphyseal distraction osteogenesis (MSDO) [64]. These techniques have been proven effective, particularly when combined with orthodontic treatment, offering successful lateral expansion as demonstrated in various case studies.

Recent research supports the predictability and efficacy of corticotomy-assisted orthodontic treatment for mandibular expansion. One of the key techniques used is selective alveolar decortication, which involves making small perforations in the cortical bone to accelerate bone turnover and expedite tooth movement. This approach not only shortens the overall treatment duration but also enhances the stability of the expansion, reducing the risk of relapse [65]. The acceleration of tissue turnover facilitates quicker and more stable outcomes, making corticotomy-assisted techniques particularly valuable in treating mandibular transverse deficiencies.

Mandibular symphyseal distraction osteogenesis (MSDO) is another minimally invasive procedure designed to expand the mandibular arch without compromising periodontal health [64-72]. The minimally invasive nature of MSDO, especially when using tooth-borne appliances such as the hyrax distractor, lies in the use of smaller incisions, local anesthesia, and fewer complications compared to traditional orthognathic surgery [64-72]. This approach promotes gradual bone formation at the osteotomy site, enhancing stability while minimizing soft tissue trauma [64-72]. Compared to more invasive methods, MSDO offers faster recovery and allows for earlier orthodontic intervention, making it a patient-friendly and effective alternative for mandibular expansion.

The success of MSDO depends heavily on the careful selection of the osteotomy site, taking into account factors such as crowding, root form, root angulations, proximity to nearby roots, the dental and skeletal midline, and bone thickness [64]. By strategically planning the osteotomy location, surgeons can achieve an effective and stable expansion. Studies have shown that stability in the posterior mandible is often better maintained compared to the anterior region, particularly when dental retention is reinforced through fixed appliances [66]. This suggests that while MSDO is effective, the anterior mandible may require additional attention during the post-operative phase to prevent relapse.

The advantages of distraction osteogenesis, particularly MSDO, are a lower risk of relapse compared to other expansion methods and its aesthetic effectiveness [64]. Because the procedure promotes natural bone formation, it offers a stable and harmonious expansion of the mandibular arch when executed with precision [64]. However, the success of mandibular expansion procedures hinges on conservative surgical planning and careful post-operative management. These will ensure that the results are not only immediate but also long-lasting.

Hierarchy of stability

In their hierarchy of stability for orthognathic surgeries, Proffit et al. [31] concluded that the most stable procedures include Le Fort I maxillary superior repositioning and forward movement of the mandible via BSSO in patients with increased anterior facial height (commonly referred to as a long face) or when anterior facial height is maintained. This combination is particularly stable because it effectively addresses both vertical and horizontal discrepancies, often with the support of rigid internal fixation in the mandible. The use of rigid fixation enhances the stability of the surgical outcomes by providing robust support to the repositioned bone segments, minimizing the risk of post-surgical movement and relapse.

However, Proffit et al. [31] also highlighted that certain movements are inherently less stable. For example, mandibular setbacks are particularly prone to instability, largely due to the inclination of the ramus, which plays a crucial role in determining the overall stability of the procedure. The inclination introduces biomechanical challenges that can lead to relapse if not carefully managed. Similarly, the downward movement of the maxilla, which results in the downward rotation of the mandible, also presents significant stability challenges. This movement disrupts the balance of forces in the masticatory system and increases the likelihood of post-operative instability. The least stable movement, according to Proffit's findings [31], is the transverse expansion of the maxilla. This instability is particularly concerning, given the common need for transverse expansion in cases of maxillary deficiency. While SARPE has been suggested as a more stable alternative to Le Fort I osteotomy for this purpose, further research is necessary to confirm its long-term efficacy and stability [31]. By facilitating more controlled expansion, SARPE may reduce the risk of relapse, but the long-term success of this technique remains contingent on careful surgical execution and post-operative care. Table 2 shows the stability hierarchy of orthognathic procedures.

**Table 2:** Hierarchy of stability of different orthognathic surgical procedures BSSO: Bilateral Sagittal Split Osteotomy, SARPE: Surgically Assisted Rapid Maxillary Expansion, MSDO: Mandibular Symphyseal Distraction Osteogenesis, PSFI: Patient-Specific Fixation Implants

Surgical procedure	Stability rating	Key factors affecting stability
Le Fort I: maxillary advancement	Moderate	Relapse due to extent of advancement, overcorrection helps mitigate relapse [45].
Le Fort I: maxillary setback	Moderate to high	Stable when IPPF (Intentional Pterygoid Plate Fracture) is used, reduced blood loss, faster recovery [46].
BSSO: mandibular advancement	High	Minimal relapse when bicortical screw fixation is used, slight movement observed [48].
BSSO: mandibular setback	Low	High relapse rate, forward relapse in the horizontal plane [51].
Maxillary impaction	Moderate	Relapse more common in bi-maxillary surgeries, vertical control is critical [56].
Maxillary inferior repositioning	Low	Significant relapse due to lack of bone grafts, instability in vertical plane [60].
SARPE: maxillary expansion	Moderate to high	Stable with minimal relapse when retention devices are used, precise surgical execution [61].
MSDO: mandibular expansion	Moderate	Effective with careful planning, anterior mandible requires extra care [64, 66].

Recent advances and prospects

Since it was first introduced in the 19th century, orthognathic surgery has undergone continuous improvements and refinements. Over the decades, it has evolved into a highly sophisticated field [67]. Traditionally, the planning of orthognathic surgery relied on cast dental models to simulate jaw movements on semi-adjustable articulators. While this approach offered a basic framework for understanding occlusal changes, it was inherently limited, particularly in addressing sagittal and vertical discrepancies. These limitations were due to the reliance on static models that failed to account for the dynamic nature of bone positioning and other anatomical interferences. Consequently, this method often emphasized occlusion over the precise alignment of skeletal structures, leading to potential inaccuracies in surgical outcomes.

The advent of virtual planning has revolutionized the field by allowing surgeons to move beyond these limitations. As a result of computer-aided manufacturing, custom splints, and guide stents have replaced traditional cast models, offering greater precision in surgical planning and execution [2,19,20]. This technology enables detailed visualization of the patient’s anatomy, allowing for more accurate jaw movement simulations and better anticipation of surgical challenges. Furthermore, the use of patient-specific implants for osteotomies has significantly enhanced the long-term stability of these procedures. These implants are tailored to the unique anatomical features of each patient. Accordingly, they provide robust support and ensure that complex cases can be treated with greater predictability, leading to higher patient satisfaction and reduced treatment durations [2,19,20].

As technology continues to advance, new developments in orthognathic surgery have emerged, further improving patient outcomes and overall satisfaction. One such advancement is the surgery-first approach, which was found by Strohl et al. [68] to eliminate the need for pre-surgical orthodontics. This approach significantly shortens the total treatment time and offers immediate improvements in facial aesthetics. The introduction of 3D printing technology is another breakthrough, enabling the creation of precise surgical models and guides that enhance the long-term stability of surgical outcomes [68]. These innovations not only enhance the efficiency of the surgical process but also improve the accuracy of the results, reducing the likelihood of post-surgical complications and relapse.

Jandali et al. [67] further supported the use of virtual planning and 3D printing within the surgery-first approach, particularly in the treatment of obstructive sleep apnea. In patients with obstructive sleep apnea, maxillomandibular advancement is considered the gold standard for managing complex airway obstructions. By incorporating advanced technologies like virtual planning and 3D printing, surgeons can achieve better surgical precision and patient outcomes [67]. The application of these technologies in maxillomandibular advancement has demonstrated their potential to revolutionize the field, particularly in complex cases where traditional methods may fall short.

In another innovative development, Kim et al. [69] introduced the concept of using machine learning to predict relapse following double jaw surgery. This approach utilizes algorithms to assess key factors such as sagittal chin projection stability (position of the pogonion on the lateral cephalogram) and the likelihood of clockwise mandibular rotation, both of which are critical for determining post-surgical stability. The study evaluated six machine learning models, with three - classification and regression trees (CART), conditional inference tree (CTRE), and random forest (RF) - showing strong predictive performance. The RF model identified ramus inclination as a significant predictor of relapse, while the CART and CTRE models revealed that a clockwise rotation of more than 1.8 degrees would likely result in a significant relapse at the pogonion [69]. This tool adds a new dimension to surgical planning by allowing surgeons to anticipate potential issues and tailor interventions to mitigate the risk of relapse, thereby enhancing the overall stability of the surgical outcomes.

Zammit et al. [70] expanded on these technological advancements by theorizing about the potential of combining mid-face and mandibular osteotomies to perform synchronized surgical movements. This approach could address the 3D functional and aesthetic issues of the maxillomandibular complex more effectively. By integrating new distraction techniques, efficient airway management strategies, and computer-assisted planning with patient-specific fixation, surgeons can now handle complex cases with greater precision. This allows for simultaneous free tissue transfer and further optimizes surgical outcomes [70]. These developments highlight the increasingly sophisticated nature of orthognathic surgery, where technological advancements are not merely adjuncts but integral to achieving the desired results.

Wang et al. [71] demonstrated the practical application of these advancements by studying the computer-aided 3D simulations and navigation in orthognathic surgery (CASNOS) protocol in 23 patients undergoing double jaw surgery for skeletal class III malocclusion. The study utilized CASNOS to perform maxillary advancement via Le Fort I osteotomy and mandibular setback via BSSO. The results showed significant improvements in deformities with minimal post-surgical displacement of the condyles, indicating the procedure's reliability and stability. For example, the rightmost lateral condylar point displacement was recorded at 1.04 ± 0.42 mm, the leftmost lateral condylar point at 1.03 ± 0.39 mm, and the leftmost medial condylar point at 0.96 ± 0.39 mm. The mean intercondylar angle before surgery was 161.61 ± 5.08°, which slightly changed to 159.28 mm post-surgery. These findings suggest that CASNOS is a reliable and stable method for performing complex orthognathic procedures in adult patients with class III malocclusion [71]. The minimal displacement observed underscores the precision and effectiveness of this advanced technology in enhancing surgical outcomes.

However, with these technological advancements come increased costs for both patients and surgeons. While 3D technology reduces hospital stay times and improves overall efficiency, it also necessitates the search for more cost-effective solutions to make these benefits accessible to a broader population. As the technology becomes more widespread, it will be crucial to balance the benefits of precision and stability with the need for affordability and accessibility, ensuring that advancements in orthognathic surgery benefit all patients.

## Conclusions

Orthognathic surgery has evolved significantly, with advancements in technology and surgical techniques improving outcomes and patient satisfaction. Procedures, such as Le Fort I osteotomy, BSSO, and SARPE, have proven to be effective in addressing various skeletal discrepancies, with varying degrees of post-surgical stability. While certain movements, such as maxillary impaction and mandibular advancement, are considered highly stable, others like mandibular setback and transverse maxillary expansion present a higher risk of relapse.

The integration of virtual planning, 3D printing, and advanced fixation techniques has enhanced both the precision and predictability of these procedures. However, the long-term stability of orthognathic surgeries remains influenced by factors such as the extent of skeletal movement, patient-specific variables, and the use of proper retention methods. Continued research, especially involving newer technologies like machine learning and patient-specific implants, will further improve the predictability and stability of orthognathic surgery outcomes.
